# Supplementary figures and images for: Cadmium toxicity on communities of ammonia-oxidizing microorganisms
Source: PeerJ. 2025 Feb 21;13:e18829. doi: 10.7717/peerj.18829 (PMC11849506; doi:10.7717/peerj.18829)

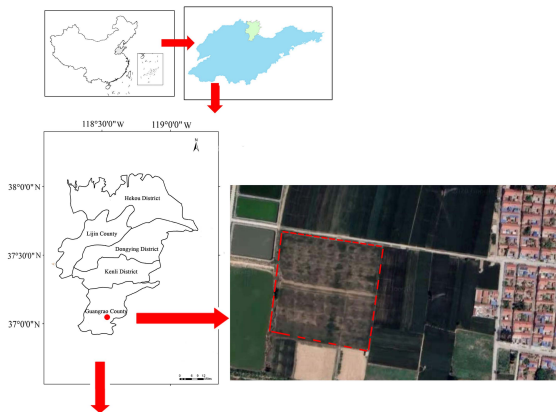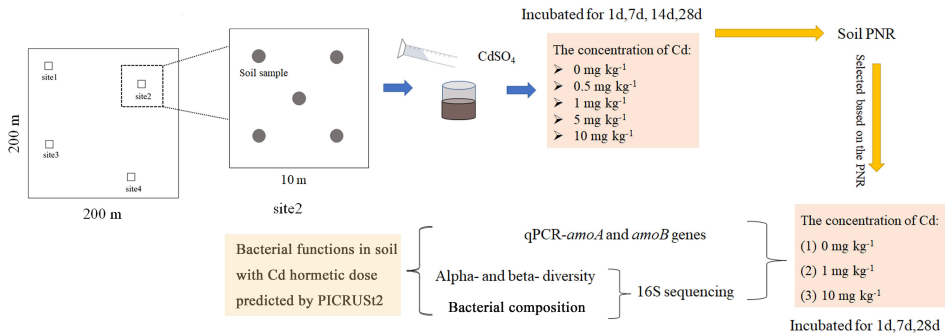

Supplement: Supplemental Information 1 — Four sampling sites in the farmland (200 m × 200 m) were randomly selected, and then, five sampling cores were used to collect soil sample for each site. These five samples were then mixed and pooled to obtain a representative sample for one sampling site. The soil samples was added CdSO4 solution at 0, 0.5, 1, 5 and 10 mg Cd kg−1 soil for incubation 1 d, 7 d 14 d and 28 d. Soil potential nitrification rate (PNR) was measured at each stage. Based on the results of PNR, soil DNA extraction, 16S sequencing and real-time fluorescence quantitative PCR (qPCR) of AOA and AOB were performed for soil samples for incubation 1, 7 and 28 d with Cd concentrations of 0, 1 and 10 mg kg−1. Finally, the PICRUSt was used to predict how Cd at the hormetic dose affects the soil bacterial functions. [file peerj-13-18829-s001.pdf]

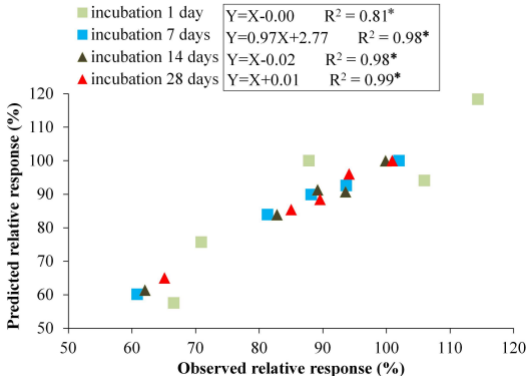

Supplement: Supplemental Information 2 — The predicted relation response values of soil PNR to Cd were obtained based on the Eq. (3) (in the ‘Soil PNR’ of manuscript), as follows, y = c + (d − c + (f∗x/exp(x)))/(1 + exp(−(x − e)/w)) (3) where y is relative response (%); x is the Cd concentration; c, d, f, e, and w are fitting parameters. Meanwhile, the linear relationship between observed relation response values of soil PNR to Cd and their predicted relation response values were expressed as the mathematical equation in the figure. [file peerj-13-18829-s002.pdf]
