# Supplementary material for: Cadmium toxicity on communities of ammonia-oxidizing microorganisms
Source: PeerJ. 2025 Feb 21;13:e18829. doi: 10.7717/peerj.18829 (PMC11849506; doi:10.7717/peerj.18829)

Dear Editors:

The original data for Figure 2 is the original data for Figure 1. Figure 2 is a chart that has been fitted based on the original data from Figure 1. Therefore, the author will also send the fitted original data (origine file) to you.


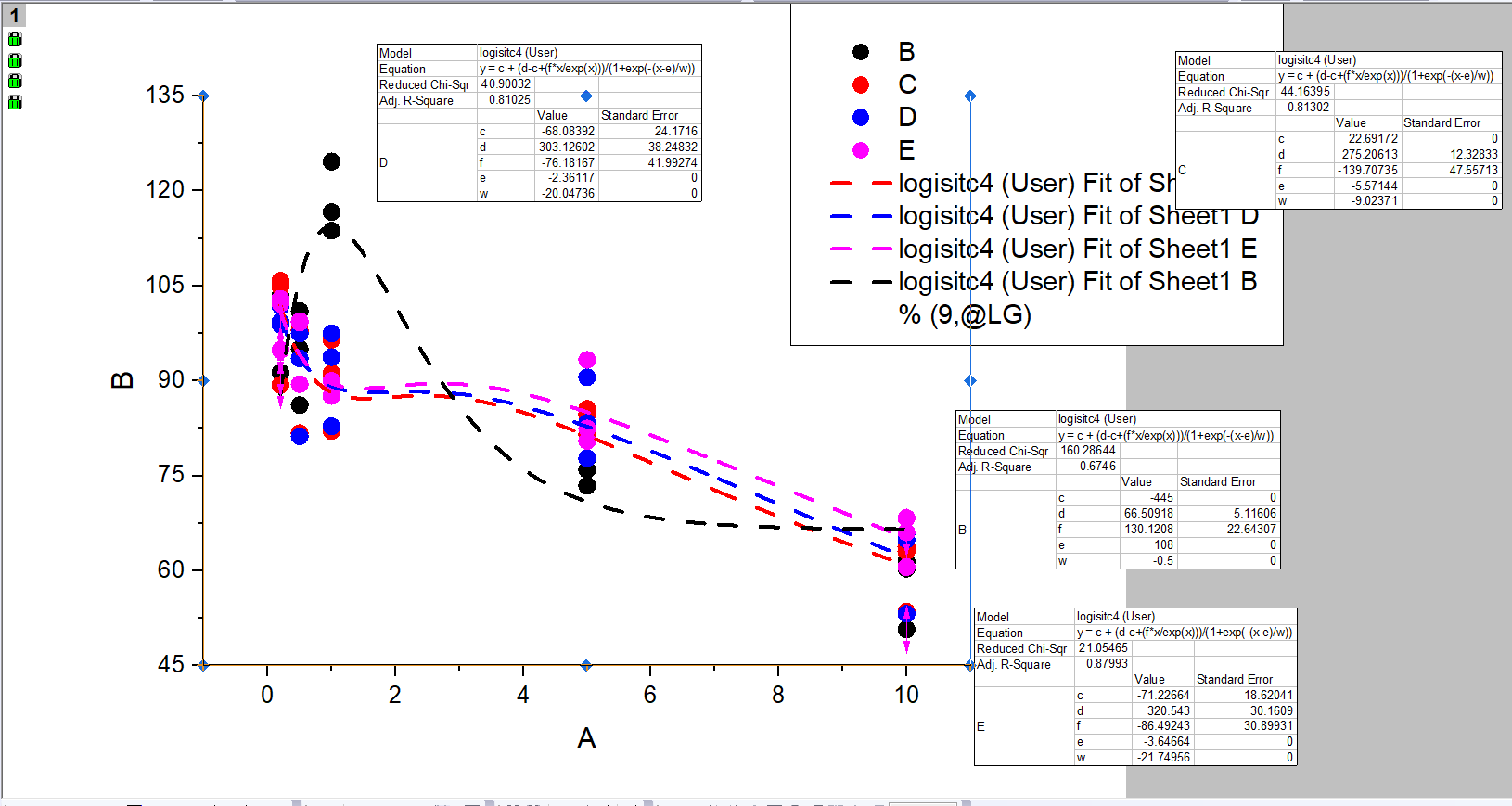

Supplement: Supplemental Information 7 [file peerj-13-18829-s007.docx]
